# Health Care Management Models for the Evolution of Hospitalization in Acute Inpatient Psychiatry Units: Comparative Quantitative Study

**DOI:** 10.2196/15776

**Published:** 2020-11-30

**Authors:** Susel Góngora Alonso, Beatriz Sainz-De-Abajo, Isabel De la Torre-Díez, Manuel Franco-Martin

**Affiliations:** 1 Department of Signal Theory and Communications, and Telematics Engineering University of Valladolid Valladolid Spain; 2 University Rio Hortega Hospital Valladolid Spain

**Keywords:** acute inpatient psychiatry unit, database, hospitalizations, mental health, readmission, SPSS

## Abstract

**Background:**

Mental health disorders are a problem that affects patients, their families, and the professionals who treat them. Hospital admissions play an important role in caring for people with these diseases due to their effect on quality of life and the high associated costs. In Spain, at the Healthcare Complex of Zamora, a new disease management model is being implemented, consisting of not admitting patients with mental diseases to the hospital. Instead, they are supervised in sheltered apartments or centers for patients with these types of disorders.

**Objective:**

The main goal of this research is to evaluate the evolution of hospital days of stay of patients with mental disorders in different hospitals in a region of Spain, to analyze the impact of the new hospital management model.

**Methods:**

For the development of this study, a database of patients with mental disorders was used, taking into account the acute inpatient psychiatry unit of 11 hospitals in a region of Spain. SPSS Statistics for Windows, version 23.0 (IBM Corp), was used to calculate statistical values related to hospital days of stay of patients. The data included are from the periods of 2005-2011 and 2012-2015.

**Results:**

After analyzing the results, regarding the days of stay in the different health care complexes for the period between 2005 and 2015, we observed that since 2012 at the Healthcare Complex of Zamora, the total number of days of stay were reduced by 64.69%. This trend is due to the implementation of a new hospital management model in this health complex.

**Conclusions:**

With the application of a new hospital management model at the Healthcare Complex of Zamora, the number of days of stay of patients with mental diseases as well as the associated hospital costs were considerably reduced.

## Introduction

Having good mental health allows us to develop the social and intellectual skills that are needed to face new challenges in everyday life [1]. The World Health Organization has determined that mental health is a cornerstone of general health. Large-scale collection of mental health–related data is difficult and is done infrequently. It is a challenge for researchers to evaluate seasonal, weekly, or diurnal trends [2].

Mental illnesses can cause mild to severe disorders in thinking and behavior; they can incapacitate patients, preventing them from carrying out the ordinary demands and routines of life [3]. Some of the most common disorders are clinical depression, bipolar disorder, dementia, schizophrenia, and anxiety disorders. The problem for people who suffer from these disorders is when specialists do not interpret the symptoms correctly. Symptoms may include changes in mood, personality, or personal habits and/or social withdrawal. Mental health problems may be related to excessive stress due to a particular situation or a series of events [4]; their prevalence is high worldwide. At least 1% of any population is incapacitated by a serious mental disorder at a specific time. The percentage of people affected in any period of their lives is 10% [1].

Hospital admissions are important events in the care of people with mental disorders due to the associated costs and their possible effect on quality of life [5]. Despite the application of various personalized treatments, the rate of relapse among the mentally ill is relatively high. It is estimated that the relapse rate among people with schizophrenia is between 50% and 92%. This implies high morbidity and high readmission rate. As a consequence, this relapse rate has a high cost to the health care system and community services [6].

In Fleury et al [7], the authors showed that only 17% of patients had received a follow-up appointment before hospital discharge. Best practices recommend brief hospitalizations and postdischarge follow-ups to improve social integration and recovery. Psychiatric care is still necessary for a small subgroup of patients who cannot be treated safely or effectively at home [8].

The majority of hospitalized psychiatric patients can be discharged without extensive follow-up. However, patients with a serious mental illness need long-term aftercare [9]. Early psychiatric readmission serves as a negative indicator of the quality of care in mental health services. Some studies report that days of stay of hospitalized patients under 28 days increase readmission rates [10].

In the Healthcare Complex of Zamora, Spain, one of the hospital centers analyzed in this work, a new management model has been applied since 2012. It consists of not admitting patients with mental diseases to the hospital. Instead, they are supervised in sheltered apartments or centers for patients with these types of disorders. Hence, the main objective of this research is to evaluate the evolution of hospital admissions and days of stay of patients with mental diseases in 11 hospital centers of a region of Spain. From the data, we can see the effects of the application of a new model on monitoring patients outside the hospital.

There are similar studies that show us the feasibility of our research. In Cooper et al [11], the authors described the service provision of 32 hospitals and evaluated the changes in the management and quality of the service, comparing it with the results of a previous study over a period of 10 years. Steeg et al [12] presented a study that applied methods of multiple imputation and propensity score. Four types of hospital management were related to patients who self-harm and a risk of suicide in these patients in the following 12 months. As a result, it was concluded that the propensity score adjustment seemed to mitigate only some of the greatest risks observed and that the differences between the treatment groups had little impact on the reduction of suicide.

Below we show the methodology used in this study, the results achieved, and, finally, the discussion and conclusions of the investigation.

## Methods

The hospital admission records for this study were extracted from an anonymized database of patients with mental diseases. The database includes a total of 53,641 records from 11 public health care complexes in Castilla and León, Spain. Once the data were processed, we included in the study the acute inpatient psychiatry unit (AIPU) of each hospital, which included a total of 49,824 admissions. The data follow the International Classification of Diseases, Ninth Revision (ICD-9), and the study period covers the years from 2005 to 2015. The data include admissions of patients with the following mental disorders: schizophrenia, bipolar disorder, Alzheimer disease, depression, autism, disorders due to drugs and alcohol, affective disorders, and other psychoses.

**Figure 1:**
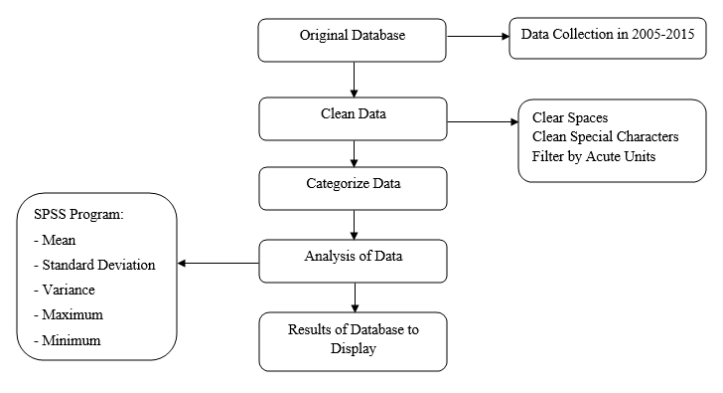
Study flowchart.

The database includes the name of the hospital, the gender of the patient, the year of admission, the number of days of stay, the date of admission, the date of discharge, the diagnosis, and the therapies used according to the diagnosis. For this study, the three selected variables were (1) the name of the hospital, (2) the days of stay, and (3) the year of admission. The rest of the variables were excluded. In addition, null values, double blanks, and special characters were removed. Figure 1 shows the flowchart followed in this study.

To obtain the descriptive and inferential statistics of the data from the 11 health care complexes during the periods 2005-2011 and 2012-2015, we used SPSS for Windows, version 23.0 (IBM Corp). We calculated the following parameters: mean, standard deviation, variance, minimum number of days of stay, and maximum number of days of stay.

## Results

For the study, 49,824 out of 53,641 (92.88%) database records from anonymous hospital admissions, from 2005 to 2015, were included. All the records were part of the AIPUs of 11 health care complexes in Castilla and León, Spain. The data obtained from the total number of days of stay per year are shown in Table 1 for Zamora, Ávila, Burgos, León, and Palencia, and in Table 2 for Salamanca, Soria, Segovia, University Clinical Hospital of Valladolid, The Bierzo Hospital, and the University Hospital of Rio Hortega.

**Table 1:** Total number of days of stay per admission year for hospital centers in Zamora, Ávila, Burgos, León, and Palencia.

Year	Days of stay per health care complex, n (%)				
	Healthcare Complex of Zamora (N=59,789)	Healthcare Complex of Ávila (N=40,842)	Healthcare Complex of Burgos (N=131,948)	Healthcare Complex of León (N=70,371)	Healthcare Complex of Palencia (N=54,395)
2005	8336 (13.94)	2511 (6.15)	13,579 (10.29)	6060 (8.61)	4883 (8.98)
2006	8012 (13.40)	2415 (5.91)	12,920 (9.79)	6033 (8.57)	4842 (8.90)
2007	5979 (10.00)	2268 (5.55)	13,509 (10.24)	6574 (9.34)	4631 (8.51)
2008	6171 (10.32)	5401 (13.23)	13,189 (9.99)	6769 (9.62)	4845 (8.91)
2009	7166 (11.99)	2648 (6.48)	12,465 (9.45)	4815 (6.84)	4799 (8.82)
2010	6953 (11.63)	3871 (9.48)	11,406 (8.64)	6874 (9.77)	5073 (9.33)
2011	7135 (11.93)	5878 (14.39)	12,860 (9.75)	6700 (9.52)	6348 (11.67)
2012	3412 (5.71)	5261 (12.88)	10,777 (8.17)	7603 (10.81)	5056 (9.29)
2013	1217 (2.04)	3802 (9.31)	11,002 (8.34)	6812 (9.68)	4677 (8.60)
2014	2051 (3.43)	3535 (8.66)	9375 (7.10)	6451 (9.17)	4584 (8.43)
2015	3357 (5.61)	3252 (7.96)	10,866 (8.24)	5680 (8.07)	4657 (8.56)
Total	59,789 (100)	40,842 (100)	131,948 (100)	70,371 (100)	54,395 (100)

**Table 2:** Total number of days of stay per admission year for hospital centers in Salamanca, Soria, Segovia, Valladolid, and El Bierzo.

Year	Days of stay per health care complex, n (%)					
	Healthcare Complex of Salamanca (N=62,031)	Healthcare Complex of Soria (N=98,242)	Healthcare Complex of Segovia (N=54,783)	University Clinical Hospital of Valladolid (N=97,303)	The Bierzo Hospital (N=44,069)	University Hospital of Rio Hortega (N=34,896)
2005	7186 (11.58)	8806 (8.96)	5470 (9.98)	7713 (7.93)	4360 (9.89)	0 (0)
2006	7171 (11.56)	8102 (8.25)	5621 (10.26)	7686 (7.90)	3849 (8.73)	0 (0)
2007	5346 (8.62)	8353 (8.50)	4488 (8.19)	8762 (9.00)	4259 (9.67)	0 (0)
2008	5910 (9.53)	9081 (9.25)	3997 (7.30)	9629 (9.90)	4679 (10.62)	0 (0)
2009	5194 (8.37)	8360 (8.51)	4852 (8.86)	8726 (8.97)	4526 (10.27)	233 (0.67)
2010	4504 (7.26)	7637 (7.77)	4460 (8.14)	9053 (9.30)	4241 (9.62)	5920 (16.96)
2011	5367 (8.65)	9354 (9.52)	4948 (9.03)	8122 (8.35)	4051 (9.19)	5239 (15.01)
2012	5477 (8.83)	9659 (9.83)	4614 (8.42)	9405 (9.66)	3920 (8.90)	5817 (16.67)
2013	6597 (10.64)	10,542 (10.73)	5366 (9.80)	9318 (9.58)	3333 (7.56)	5873 (16.83)
2014	4899 (7.90)	10,482 (10.67)	5425 (9.90)	9676 (9.94)	3494 (7.93)	5784 (16.58)
2015	4380 (7.06)	7866 (8.01)	5542 (10.12)	9213 (9.47)	3357 (7.62)	6030 (17.28)
Total	62,031 (100)	98,242 (100)	54,783 (100)	97,303 (100)	44,069 (100)	34,896 (100)

The results show that the Healthcare Complex of Burgos had the highest number of days of stay during the 11 years analyzed; it represents 17.62% (131,948/748,669) of the total sample. The Healthcare Complex of Soria had the next highest total number of days of stay, with 13.12% (98,242/748,669) of the total sample. The University Hospital of Rio Hortega in Valladolid had the lowest number of days of stay of 4.66% (34,896/748,669) of the total, but we must consider that the data have only been recorded for this hospital since 2009. The Healthcare Complex of Ávila represents 5.46% (40,842/748,669) of the total sample of days of stay. In 2012, a new hospital management model was implemented at the Healthcare Complex of Zamora; under this model, patients are supervised in sheltered homes or other similar centers. Tables 1 and 2 show the decrease in the number of patient days of stay in that hospital.

Through the SPSS statistics program, we obtained the following statistical parameters: mean, standard deviation, variance, minimum, and maximum, taking into account the total number of days of stay per hospital complex in the periods 2005-2011 and 2012-2015 (see Table 3).

**Table 3:** Descriptive statistics of total days of stay per hospital over 11 years.

Health care complex and years		Days of stay			
		Mean (SD)	Variance	Minimum	Maximum
**Healthcare Complex of Zamora**					
	2005-2011	7107.43 (866.60)	750,987.62	5979	8336
	2012-2015	2509.25 (1066.70)	1,137,846.92	1217	3412
**Healthcare Complex of Ávila**					
	2005-2011	3570.29 (1514.97)	2,295,136.57	2268	5878
	2012-2015	3962.50 (894.32)	799,809.67	3252	5261
**Healthcare Complex of Burgos**					
	2005-2011	12,846.86 (743.74)	553,155.81	11,406	13,579
	2012-2015	10,505.00 (758.99)	576,071.33	9375	11,002
**Healthcare Complex of León**					
	2005-2011	6260.71 (719.70)	517,970.57	4815	6874
	2012-2015	6636.50 (798.80)	638,075.00	5680	7603
**Healthcare Complex of Palencia**					
	2005-2011	5060.14 (582.57)	339,392.14	4631	6348
	2012-2015	4743.50 (212.13)	45,000.33	4584	5056
**Healthcare Complex of Salamanca**					
	2005-2011	5811.14 (1020.81)	1,042,054.14	4504	7186
	2012-2015	5338.25 (951.30)	904,962.25	4380	6597
**Healthcare Complex of Soria**					
	2005-2011	8527.57 (591.55)	349,928.95	7637	9354
	2012-2015	9637.25 (1247.66)	1,556,658.25	7866	10,542
**Healthcare Complex of Segovia**					
	2005-2011	4833.71 (576.97)	332,890.91	3997	5621
	2012-2015	5236.75 (421.56)	177,712.92	4614	5542
**University Clinical Hospital of Valladolid**					
	2005-2011	8527.29 (721.09)	519,967.91	7686	9629
	2012-2015	9403.00 (198.21)	39,286.00	9213	9676
**The Bierzo Hospital**					
	2005-2011	4280.71 (278.77)	77,712.91	3849	4679
	2012-2015	3526.00 (272.07)	74,023.33	3333	3920
**University Hospital of Rio Hortega**					
	2005-2011	3797.33 (3105.53)	9,644,294.33	233	5920
	2012-2015	5876.00 (109.04)	11,890.00	5784	6030

Table 4 shows a comparison of total number of days of stay in 2012 for each hospital in relation to the mean days of stay in the period from 2005 to 2011. In the case of the University Hospital of Rio Hortega, the mean is calculated from data between 2009 and 2011, because in the previous years there were no records. The results show that since 2012, the number of days of stay in the Healthcare Complex of Zamora decreased considerably. In other health care complexes, the days of stay increase in some cases and in others the behavior is not too variable.

**Table 4:** Mean days of stay in the period from 2005 to 2011 compared to the total days of stay from the year 2012 for each hospital.

Health care complex	Days of stay from 2005 to 2011, mean (SD)	Total days of stay in 2012, n
Healthcare Complex of Zamora	7107.43 (866.60)	3412
Healthcare Complex of Ávila	3570.29 (1514.97)	5261
Healthcare Complex of Burgos	12,846.86 (743.74)	10,777
Healthcare Complex of León	6260.71 (719.70)	7603
Healthcare Complex of Palencia	5060.14 (582.57)	5056
Healthcare Complex of Salamanca	5811.14 (1020.81)	5477
Healthcare Complex of Soria	8527.57 (591.55)	9659
Healthcare Complex of Segovia	4833.71 (576.97)	4614
University Clinical Hospital of Valladolid	8527.29 (721.09)	9405
The Bierzo Hospital	4280.71 (278.77)	3920
University Hospital of Rio Hortega	3797.33 (3105.53)	5817

Table 5 reports the percentage increase and decrease in the number of days of stay for each hospital in 2012 with respect to the mean days of stay in the period from 2005 to 2011. The records for the University Hospital of Rio Hortega are limited to the years between 2009 and 2011. The results show that the percentage decrease in the number of days of stay is 52% higher than in the rest of the care complexes.

**Table 5:** Percentage increase and decrease in the number of days of stay in 2012 with respect to the mean days of stay from 2005 to 2011.

Health care complex		Increase or decrease in days of stay, %	
**Increase**			
	Healthcare Complex of Ávila		47.36
	Healthcare Complex of León		21.44
	Healthcare Complex of Soria		13.27
	University Clinical Hospital of Valladolid		10.29
	University Hospital of Rio Hortega		53.19
**Decrease**			
	Healthcare Complex of Zamora		52.00
	Healthcare Complex of Burgos		16.11
	Healthcare Complex of Palencia		0.08
	Healthcare Complex of Salamanca		5.75
	Healthcare Complex of Segovia		4.55
	The Bierzo Hospital		8.43

The results from Tables 4 and 5 show how the days of stay at the Healthcare Complex of Zamora decreased considerably in 2012, with respect to other hospital centers. In Table 6, we show the evolution of the number of days of stay in this hospital from 2012 to 2015, with respect to the mean days of stay from 2005 to 2011.

**Table 6:** Percentage decrease in the number of days of stay for each year from 2012 to 2015 with respect to the mean days of stay from 2005 to 2011 for the Healthcare Complex of Zamora.

Year	Days of stay
2005-2011, mean (SD)	7107.43 (866.60)
2012, % decrease	52.00
2013, % decrease	82.88
2014, % decrease	71.15
2015, % decrease	52.77

## Discussion

Once a serious mental illness occurs it tends to become chronic, and a patient may need repeated hospitalizations that affect daily life and social integration. Therefore, early diagnosis, proper treatment, and follow-up of mental health disorders are crucial for disease prevention [13].

In this study, we used a total of 49,824 records of anonymous hospital admissions of patients with mental diseases. The sample corresponds to a time period of 11 years, from 2005 to 2015, and includes 11 AIPUs.

It is necessary to highlight the variation of the behavior of days of stay per year in the Healthcare Complex of Zamora since 2012; in Tables 1, 2, and 3, the percentage of days of stay of this hospital compared to other hospitals reduced the mean number of days of stay by 64.69%.

This favorable variation of the total number of days of stay per year is due to the hospital management model that was implemented; that is, patients with mental diseases do not go to the hospital for treatment. Instead, the specialists travel to the primary health care center to see their patients. This allows for the integration of levels of care in primary and hospital care. The sheltered homes are another aspect of the applied management model, where the patient feels integrated into society and their daily lives.

Since 2012, in the Healthcare Complex of Ávila and the University Hospital of Rio Hortega, the percentage of days of stay with respect to the mean days of stay from 2005 to 2011 has increased by 47.36% and 53.19%, respectively (see Tables 4 and 5). Table 5 indicates that in the Healthcare Complex of Burgos and The Bierzo Hospital, the days of stay decreased by 16.11% and 8.43%, respectively. These values are lower compared to the value of days of stay in the Hospital of Zamora in 2012 (n=3412), which represents a decreased mean of 52.00% from the previous years (mean 7107.43, SD 866.60).

Table 6 shows the decrease in the number of days of stay for patients with mental diseases since 2012. In 2013, the highest decrease of 82.88% was observed, which represents a total of 1217 days of stay registered in that year.

In relation to expenses, a stay in these health care complexes costs between €325 and €408 (US $384.71 and US $482.96) [14]. Taking into account the Healthcare Complex of Zamora, the mean number of days of stay between 2005 and 2011 represents a cost of €2,309,914.75 to €2,999,731 (US $2,734,280.74 to US $3,550,826.58). When applying the hospital management model, the cost of days of stay was reduced in 2012 by 51.99%; it corresponds to a value of €1,108,900 to €1,392,096 (US $1,312,621.56 to US $1,647,844.92). Therefore, in the 2012-2015 period, the cost of the mean days of stay was reduced by 64.69%.

These results allow us to demonstrate the efficiency of the management model applied in the Healthcare Complex in Zamora. Hence, we propose the following as future lines of study: (1) apply the hospital management model to the rest of the health care complexes to improve management efficiency, (2) analyze the results in subsequent years using the same model, comparing it with what was obtained previously, (3) analyze the trend of mental health diseases in the data set and determine the main disorders in this region, and (4) apply machine learning techniques to the database in order to obtain predictions of the most prevalent mental disorders in patients.

## Abbreviations

AIPU: acute inpatient psychiatry unit
ICD-9: International Classification of Diseases, Ninth Revision
